# The effect of abandonment on vegetation composition and soil properties in *Molinion* meadows (SW Poland)

**DOI:** 10.1371/journal.pone.0197363

**Published:** 2018-05-17

**Authors:** Grzegorz Swacha, Zoltán Botta-Dukát, Zygmunt Kącki, Daniel Pruchniewicz, Ludwik Żołnierz

**Affiliations:** 1 Botanical Garden, University of Wrocław, Wrocław, Poland; 2 MTA Centre for Ecological Research, Institute of Ecology and Botany, Vácrátót, Hungary; 3 Department of Botany and Plant Ecology, Wrocław University of Environmental and Life Sciences, Wrocław, Poland; RMIT University, AUSTRALIA

## Abstract

Intermittently wet meadows of the *Molinion* alliance, as with many other grasslands of high-nature value, have become increasingly exposed to abandonment due to their low economic value. The potential consequences of land abandonment are the decrease in species diversity and environmental alterations. The issue of land-use induced changes in plant species composition and soil physico-chemical parameters have been rarely studied in species-rich intermittently wet grasslands. In this study we attempt to i) to identify determinants of plant species composition patterns and ii) to investigate the effect of cessation of mowing on vegetation composition and soil properties. The study was conducted in an area of 36 ha covered with *Molinion* meadows, comprising of mown sites and sites that were left unmown for 10 years. In total, 120 and 80 vegetation plots were sampled from mown and unmown sites, respectively. In these plots we measured plant community composition and soil physico-chemical parameters. The results have shown that the two groups of variables (soil properties and management) differ considerably in their ability to explain variation in plant species data. Soil variables explained four-fold more variation in plant species composition than management did. The content of soil organic matter, moisture, total nitrogen and exchangeable forms of potassium, calcium and magnesium were significantly higher in mown than in unmown grassland systems. The results revealed that soil organic matter was the component of the soil most strongly affected by management, followed by moisture, magnesium, calcium and potassium in that order. Each of these soil parameters was negatively correlated with the abundances of woody plants and invasive species. We concluded that low-intensity, late time of mowing is suitable grassland management practice to ensure high plant species diversity and sustainability of the grassland ecological system while cessation of mowing not only lead to reduced plant species richness and diversity, but also to reduced nutrient levels in grassland soils.

## Introduction

Semi-natural grasslands are among the most endangered ecosystems in Europe owing to alterations in land management [1]. Grasslands have traditionally been managed by mowing or grazing, or alternatively by the application of these two management systems simultaneously [2]. However, since the late 20th century, semi-natural grasslands have been losing their economic importance as a consequence of decreased demand for hay and smaller numbers of livestock. Consequently, vast areas of agricultural land in Europe, including grasslands, have been abandoned [3,4]. The remaining high-diversity grasslands are often protected by statutory designations that require extensive management regimes. Species-rich intermittently wet meadows are an example of the habitats thus protected. These meadows support many rare species and high floristic diversity, and as a result, are included in the Natura 2000 network. In addition, they are often protected under EU-based agri-environmental scheme agreements that are designed to maintain the farming practices under where these specific vegetation types have developed.

A host of studies have reported that semi-natural grasslands are vulnerable to changes in management practises [2,5,6]. These particularly refer to the ecologically balanced grassland ecosystems in which ecological processes generally play a key role in regulating species composition and structure of vegetation. Extensive mowing enables the existence of plant species adapted to different levels of biomass removal, thus generating high species diversity. In contrast, cessation of management in semi-natural grasslands triggers secondary succession and leads to progressive changes in species composition and the structure of the vegetation stand. Consequently, cessation of suitable agricultural management results in succession towards shrub-dominated communities or, at longer time-scales, to tree-dominated communities [7]. Lack of management of semi-natural grasslands reduces species diversity through competitive exclusion [8], reduced light availability and litter accumulation suppressing recruitment of seedlings [9]. Moreover, early-successional grasslands are often invaded by highly competitive alien species that outcompete the native components of vegetation [10].

Species composition in grasslands is determined by complex ecological processes that involve both biotic and abiotic factors [11,12]. Studies comparing the relative importance of abiotic conditions and management practises present conflicting views regarding which factors most strongly impact plant species composition. On one hand, site-specific soil factors are indicated as the most important predictors of species composition [13–15], while other reports suggest greater importance of current management practises [16]. Therefore, it is essential to clarify the relative impact of environmental and management factors on species composition in different grassland ecosystems, as well as to assess the combined effects of these factors. Little is known about the interactions between soil and management factors on the species composition and soils physico-chemical properties in grassland systems. An important question that remains unanswered is whether successional changes in species composition are reflected by changes in soil properties [17–19]. The issue of changes in grassland soils has been widely studied, particularly regarding the impact of grazing intensity and fertilisation on nutrient cycling [20], as well as the effect of different mowing regimes on soil physico-chemical properties [2]. Long-term mowing is often expected to decrease the availability of nutrients in the soil, particularly potassium [21], nitrogen and phosphorus [22,23], owing to the export of nutrients with the harvested biomass. On the other hand, nutrient impoverishment in grassland soils due to biomass extraction has not been found by other researchers [24]. Conflicting findings from different studies suggest that nutrient recycling processes are case dependent and may be strongly related to vegetation type, past land use and pedoclimate. The effects of land abandonment on soil physico-chemical properties have not been sufficiently examined thus far, especially in extensively and late-harvested lowland grassland systems.

The aim of this study is two-fold. In the first step, we attempt to investigate the relative impact of management and soil physico-chemical properties on plant species composition patterns. Henceforth, every mention of the word management refers to mown versus unmown. In the second step, we attempt to investigate the effect of cessation of mowing on vegetation composition, diversity and richness in *Molinion* meadows. We hypothesised that soil properties and management significantly influence species composition patterns and have a strong shared effect. The causality between vegetation composition, soil properties and management was assessed using the variation partitioning method [25]. We also put forward the hypothesis that cessation of mowing influences soil physico-chemical properties through succession-related changes in vegetation composition during the early stage of succession. To test our hypotheses, we analysed meadow sites adjacent to each other containing the same vegetation type but with differing management practises. Two different management patterns were differentiated within the study area based on previous long-term observations of the study area: i) meadows mown once every two years towards the end of summer and ii) sites abandoned for 10 years with substantial successional changes [26]. The underlying assumption is that these sites were originally uniform in terms of species composition patterns and soil properties and the expected differences are solely due to abandonment. As it has been stressed in the literature, a comparative study design is a good tool for evaluating temporal changes in vegetation composition and structure, given that the compared sites represent subsequent stages in a temporal series of the same habitat type [27].

## Materials and methods

### Study area

**Fig 1:**
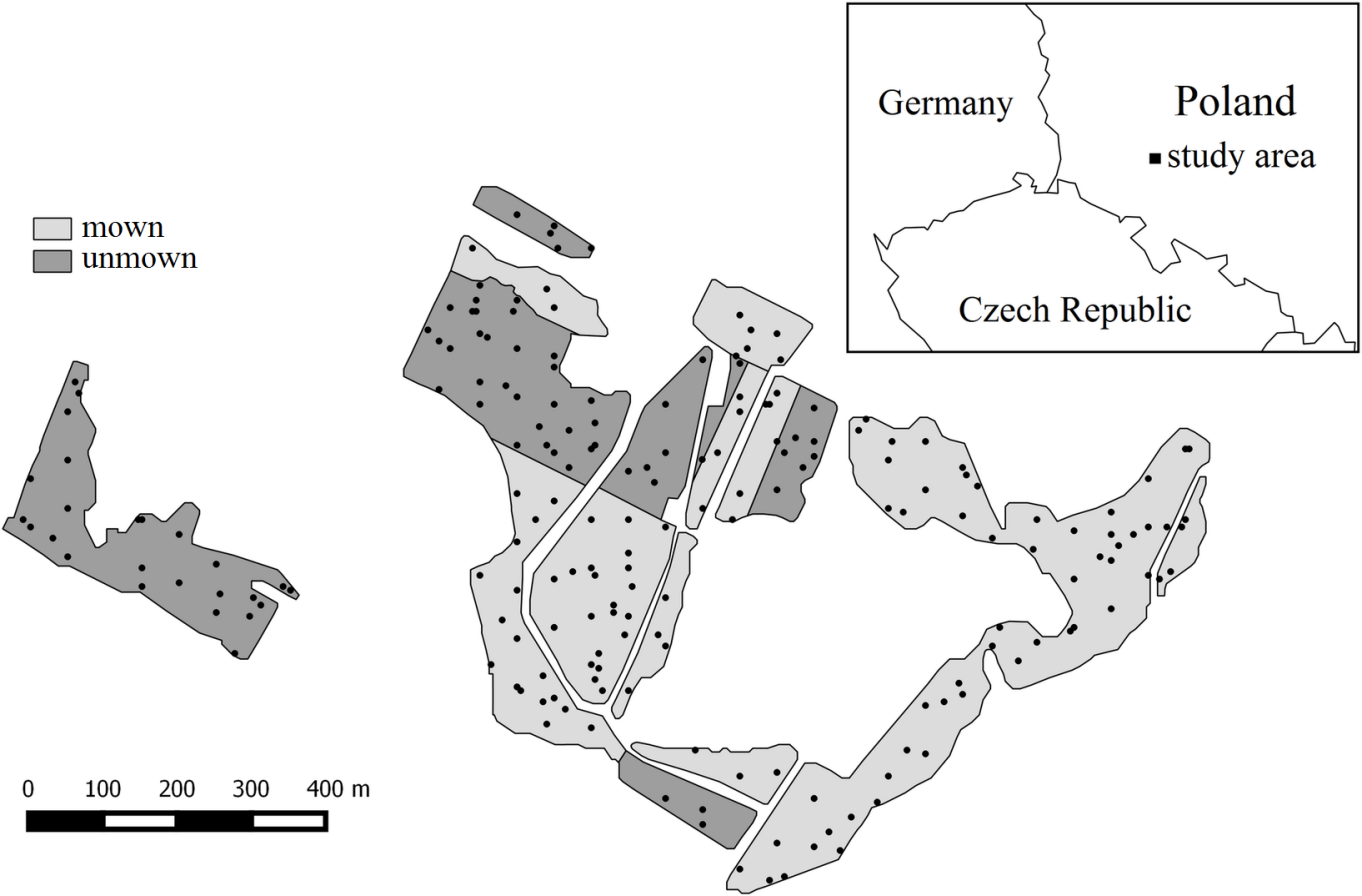
Distribution of sampling plots in the study area.

The study area is in south-western Poland in the Natura 2000 site ‘Zagórzyckie Łąki’ (N 51°15′24,8″, E 16°33′24,9″) (Fig 1). It is situated in the lowlands with an elevation range of 105–125 m above sea level. The average annual precipitation is 600 mm, with a mean annual temperature of 8.5° C [28]. The study site comprises 36 ha of permanent grasslands covered with vegetation of the alliance *Molinion caeruleae* Koch 1926, which corresponds to Natura 2000 habitat type 6410. Two types of management patterns were differentiated within the study area based on long-term observations of the study area. More than half of the study area (ca. 60%) is currently managed by mowing once every two years towards the end of summer without application of fertilizers or manure. The other parts are no longer managed and have been undergoing secondary succession for about 10 years. The entire area was homogenous regarding vegetation type prior to land abandonment. Even after ten years of abandonment, vegetation found in unmown sites bear a strong floristic resemblance to the adjacent mown meadows [26]. These meadows had been used for hay making for several decades before the abandonment. The dominant species for mown sites, in descending order according to their cover, were *Calamagrostis epigejos*, *Molinia caerulea*, *Galium boreale*, *Festuca rubra*, *Sanguisorba officinalis*, *Filipendula ulmaria*, *Stachys officinalis*, *Carex acutiformis*, *Ranunculus repens* and *Alopecurus pratensis*. The dominant species for unmown sites were *Solidago gigantea*, *Molinia caerulea*, *Alopecurus pratensis*, *Festuca rubra*, *Calamagrostis epigejos*, *Filipendula ulmaria*, *Galium boreale*, *Stachys officinalis*, *Frangula alnus* (shrub), and *Carex acutiformis*. Full list of species and their frequency and mean cover in mown and unmown sites is given in S1 Table.

### Vegetation sampling

Vegetation data on vascular plants was obtained from 5 x 5 m plots selected randomly (97 plots) and by systematic sampling (103 plots) with a fixed distance between plots: 65 m in the north-south gradient and 50 m in the east-west gradient. The combined sampling design made it possible to record a large gradient of environmental conditions and vegetation variability in the studied meadow complex and mitigate against the limitations of these two sampling methods [26,29]. The Braun-Blanquet seven-point scale was used for visual estimation of plant cover in each plot [30]. In total, 200 plots were sampled, 120 in the mown area and 80 in the unmown area.

### Soil sampling and chemical analyses

To characterise soil chemical parameters, we collected five subsamples of topsoil with a 25-mm diameter soil auger from each of our 200 plots at a depth of 10-cm following the removal of the upper undecomposed layers. One core was taken at each corner and one core in the middle of the plot. The subsamples from each plot were then mixed to form a bulk sample for chemical analysis. Soil samples were air-dried and sifted through a 2-mm-mesh sieve before chemical analysis. Physico-chemical analyses were performed in accordance with the methods proposed by Allen [31] and Radojević and Bashkin [32]. Loss-on-ignition as a rough measure of soil organic matter (SOM) was determined by igniting 2g of soil in a muffle furnace at 600°C for 6 hours and then cooling overnight. Soil pH was determined potentiometrically in distilled water. The content of total nitrogen (N) was determined by the Kjeldahl method. The presence of soluble phosphorus (P) was determined colorimetrically after extracting the soil with 0.5 M sodium bicarbonate solution (pH 8.5). Exchangeable forms of potassium (K), calcium (Ca) and magnesium (Mg) were extracted with 1M ammonium acetate (pH 7.0) and determined using a spectrometer (Varian SpectrAA 200) operating in the emission mode for K and Ca and atomic absorption mode for Mg. Moisture content in the soil was measured using a portable LB-797 moisture meter (LAB-EL Electronics Laboratory, Poland) in August, which is in the dry season, on a day when no rain had fallen for at least seven days prior, which has been evaluated by checking the weather on a daily basis. The measured moisture content thus represents close to the maximum dryness of the soil. Five moisture measurements were taken from the topsoil of each plot and the obtained values were averaged to express the percentage moisture of soil for a given site. Our measurements of moisture content provided only a snapshot of soil humidity, as a result we additionally calculated the average values for moisture based on Ellenberg indicator values (EIVm) for each plot [33], which integrate both groundwater level and soil moisture content [34].

### Data analysis

We used a mid-percentage value on the seven-point Braun-Blanquet scale for the further analyses [30]. The non-parametric Mann–Whitney U test was used to analyse the differences in vegetation attributes between mown and unmown sites (the number of permutation was set to 999). These attributes included species richness (per sample unit), alpha diversity (expressed according to the Shannon–Wiener index), and qualitative and quantitative proportions of plant functional groups. The functional groups were i) monocots, ii) dicots, iii) woody species (>0.5 m in height) and iv) invasive species. The non-parametric Mann–Whitney U tests was used to overcome problems of non-normal distributions. In the analysis of invasive species, we included only *Solidago gigantea*. Other invasive species were very scarce at the study site and were therefore excluded from the analysis.

To analyse the impact of soil and management factors on species composition we used redundancy analysis (RDA). Prior to performing the analysis, potential explanatory variables were tested for collinearity [35]. Based on the outcome of this test, all explanatory variables were used in the analysis. Percentage cover values of plant species were transformed using the Hellinger method [36]. Additionally, rare species (i.e. species with frequency less than 5% in the whole data set) were excluded from ordination analysis as rare species may have excessive influences on ordination results. We quantified the relative impact of soil properties and management practices on species composition using the variation partitioning method [25]. The significance of each term in the model was tested using the randomisation test with 999 permutations, but excluding the shared effect that cannot be tested for significance [37]. Adjusted coefficient of determination (R^2^_adj_) was used as the measure of the ratio of the explained variation to the total variation. The variation partitioning method enables the decomposition of variation into different variables or groups of variables i) direct (independent) effect, ii) as well as the estimation of the shared effect. The degree of variation in species composition that each soil variable independently explained was determined by partialling out the effects of all other soil variables. For testing the direct effect of a single soil variable (for example pH) on species composition we excluded all the other soil variables from the model and used pH as predictor and management practise co-variable. The same logic was applied to test direct effect of management where it was used as predictor and pH as co-variable. The direct effect of soil and management factors was then analysed for all variables of the same category.

For a more specific insight into how abandonment changed soil physico-chemical properties, we tested whether pH, moisture, SOM, N, P, K, Ca and Mg differed between mown and unmown sites. The values for soil variables deviated from the normal distribution in most cases, therefore nonparametric methods were used. For a comparison of medians, the Mann–Whitney U test was applied (with 999 permutations). Separate general linear models were constructed to analyse the impact of management practises on soil physico-chemical properties with a single soil parameter as dependent variable and management as predictor. The significance of the effects was tested by Monte Carlo permutation (with 999 permutations). In order to find components of vegetation related to changes in soil physico-chemical parameters we used Spearman-rank correlation where the correlation between abundance (percentage cover) of selected functional plant groups and the content of measured soil parameters was calculated. All statistical analyses were performed in R statistical software (http://www.r-project.org). The ordination biplot of RDA was visualized by Canoco 5 [38]. The nomenclature of taxa follows Euro+Med PlantBase (http://ww2.bgbm.org/EuroPlusMed/).

## Results

The study site hosts a high diversity of vascular plant species. In total, 250 plant species were recorded in the study area, of which 180 were dicots and 70 were monocots. More specifically, 220 (153 dicots and 67 monocots) species and 206 (151 dicots and 55 monocots) species were found in mown and unmown sites, respectively. The numbers of species per plot, as well as the Shannon–Wiener index, were significantly higher in mown sites than in unmown sites (Table 1). Mown sites had also significantly higher numbers and higher mean cover levels of monocotyledon and dicotyledonous species than in unmown sites, but lower numbers and lower mean cover levels of woody species and invasive species (*Solidago gigantea*). Abundances of woody species and invasive species were the variables most strongly affected by abandonment, with more than four-fold increase in mean cover.

**Table 1:** Comparison of vegetation parameters estimated from mown and unmown sites.

	mown (mean ± SD)	unmown (mean ± SD)	Mann–Whitney U test (*p*-value)
Number of species	39.6 ± 8.6	34.5 ± 9.7	0.001
Shannon-Wiener	3.0 ± 0.4	2.7 ± 0.5	0.001
Monocots number	13.0 ± 4.2	10.6 ± 4.8	<0.001
Monocots cover (%)	55.7 ± 16.2	48.4 ± 19.3	0.007
Dicots number^1^	25.8 ± 6.0	22.1 ± 6.1	<0.001
Dicots cover^1^ (%)	60.6 ± 13.7	50.9 ± 15.6	<0.001
Woody number^2^	0.7 ± 0.9	1.8 ± 1.6	<0.001
Woody cover^2^ (%)	2.7 ± 7.0	12.9 ± 20.6	<0.001
*Solidago* cover (%)	3.3 ± 9.3	14.2 ± 24.4	0.001

^1^excluding woody species in shrub and tree layer

^2^excluding shrubs and trees in herb layer (seedlings and juveniles).

**Fig 2:**
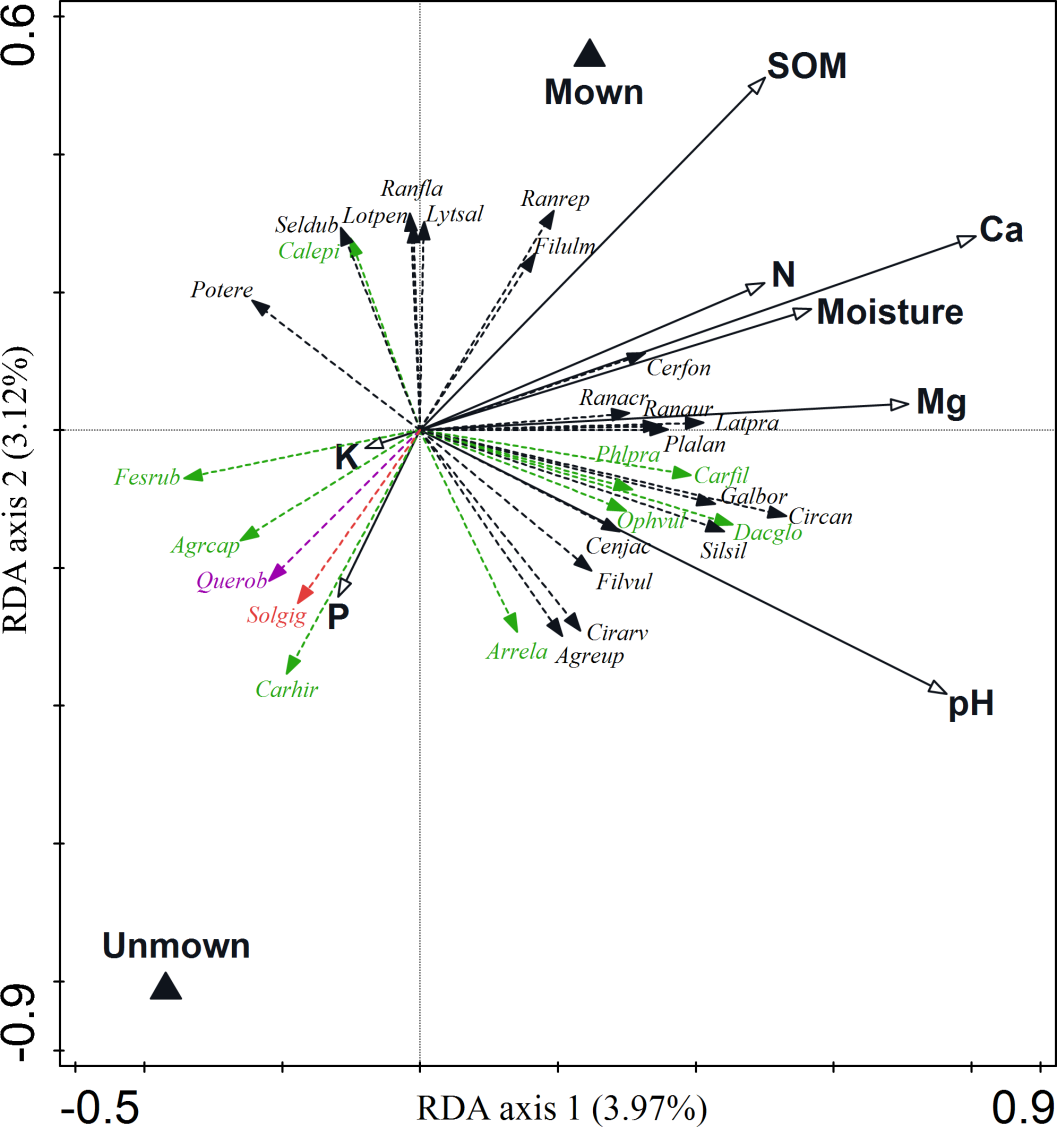
Ordination biplot of redundancy analysis (RDA) representing shared effects of two groups of explanatory variables (soil properties and management). Species data were related to the management (triangles) and soil variables (arrows). 25 best fitted species are plotted. The portion of variance explained by the respective axis is given in the axis title. See Table 2. for detailed results of the variation partitioning. Red coloured species (invasive species); purple coloured species (woody species); green coloured species (monocots), black coloured species (dicots). Species labels: Agrcap = *Agrostis capillaris*, Agreup = *Agrimonia eupatoria*, Arrela = *Arrhenatherum elatius*, Carfil = *Carex filiformis*, Carhir = *Carex hirta*, Cenjac = *Centaurea jacea*, Cerfon = *Cerastium fontanum* subsp. *vulgare*, Cirarv = *Cirsium arvense*, Circan = *Cirsium canum*, Cnidub = *Cnidium dubium*, Dacglo = *Dactylis glomerata*, Fesrub = *Festuca rubra*, Filvul = *Filipendula vulgaris*, Galbor = *Galium boreale*, Latpra = *Lathyrus pratensis*, Lotuli = *Lotus uliginosus*, Ophvul = *Ophioglossum vulgatum*, Phlpra = *Phleum pratense*, Plalan = *Plantago lanceolata*, Potere = *Potentilla erecta*, Querrob = *Quercus robur*, Ranaur = *Ranunculus auricomus*, Ranrep = *Ranunculus repens*, Silsil = *Silaum silaus*, Solgig = *Solidago gigantea*.

The two groups of explanatory variables (soil properties and management) included in the RDA model accounted for 10% of the total explained variation in species composition. Partitioning the variation of compositional data revealed that the largest fraction was attributable to the variation in soil variables, which explained about four times as much variation in species composition than management did (Table 2). The shared effect of soil and management variables accounted for 0.19% of the total variation in species composition (Table 2). The RDA model revealed that each individual soil factor significantly affected species composition after partialling out the effects of other variables. The variation in species composition was best explained by Ca and pH, followed by Mg, SOM, moisture, K, N and P. After controlling for the effects of individual soil variables, only pH and Ca had higher independent explanatory power than the independent effect of management. In all other cases, the direct effect of management on species composition was stronger than that of any single soil variable. The impact of soil parameters and management on species composition was visualized on the RDA ordination diagram representing shared effects of both explanatory groups of variables (Fig 2).

**Table 2:** Variation partitioning among direct and shared effects of soil variables and management explaining variation in species composition.

soil variables	adjusted coefficient of determination R^2^_adj_ (%) for		
	direct effect of soil	shared effect	direct effect of management
pH	2.69**	0.00	2.23**
Moisture	1.81**	0.00	2.16**
SOM	1.85**	0.21	1.88**
N	0.80**	0.18	1.91**
P	0.36*	0.05	2.04**
K	1.04**	0.00	2.34**
Ca	2.71**	0.17	1.92**
Mg	1.80**	0.10	1.99**
All variables	7.91**	0.19	1.90**

Significance codes

* p≤0.05

** p≤0.001.

Significant differences in the content of moisture, SOM, N, K, Ca and Mg were found between mown and unmown sites (Table 3). The contents of these soil parameters were significantly higher in mown than in unmown sites, except pH and P. Although there was a significant difference in measured contents of moisture, the Mann–Whitney U test did not reveal differences in moisture calculated from species composition using EIVm (EIVm for mown sites = 6.2; EIVm for unmown sites = 6.1; p = 0.122). The RDA model revealed that changes in soil parameters are, to a large extent, attributable to the management. Of the soil physico-chemical parameters, SOM was the most affected parameter followed by moisture, Mg, Ca, K and N.

**Table 3:** Comparison of soil physico-chemical parameters between mown and unmown sites and the effect of management on soil parameters.

Soil parameters	i) Differences in soil parameters			ii) Effect of management on soil parameters	
	mown (mean ± SD)	unmown (mean ± SD)	*p*-value (Mann–Whitney U test)	R^2^_adj_ (%)	*p*-value (general linear model)
pH	5.4 ± 0.5	5.5 ± 0.5	0.097	1.12	0.078
Moisture %	18.1 ± 5.3	14.7 ± 3.9	<0.001	10.30	0.001
SOM %	10.0 ± 3.6	7.1 ± 2.0	<0.001	17.42	0.001
N total %	0.4 ± 0.2	0.3 ± 0.2	<0.001	4.04	0.003
P mg kg^-1^	13.5 ± 4.5	13.9 ± 5.5	0.733	0.00	0.587
K mg kg^-1^	80.3 ± 41.7	61.7 ± 28.1	<0.001	5.35	0.001
Ca mg kg^-1^	1456.4 ± 792.2	1080.5 ± 467.5	<0.001	6.40	0.001
Mg mg kg^-1^	511.7 ± 281.1	350.5 ± 152.4	<0.001	9.54	0.001

i) Differences in soil parameters between mown and unmown sites tested by Mann–Whitney U test. ii) The effect of management on soil parameters was explored by general linear model.

The content of SOM was negatively correlated with the abundance of woody species (Table 4). A significant negative correlation was also found between the abundance of this plant functional group and other soil parameters. A statistically significant but weak negative Spearman rank correlation was found between the abundance of *Solidago gigantea* and soil properties.

**Table 4:** Relationships (Spearman rank correlations) between abundances of functional species groups and soil parameters.

	SOM	Moisture	N	K	Ca	Mg
Woody plants	-0.459***	-0.319***	-0.400***	-0.162*	-0.432***	-0.376***
*Solidago gigantea*	-0.185**	-0.141*	-0.141*	-0.020 n.s.	-0.169*	-0.146*

The asterisks denote probability levels

* p<0.05

** p<0.01

*** p<0.001; n.s., not significant.

## Discussion

### Changes in vegetation characteristics

Our study shows that a 10-year cessation of mowing leads to substantial changes in quantitative proportions of plant functional groups, diversity expressed as Shannon–Wiener index, and species richness per sampling unit, yet the total number of species (the pooled number of species) was not strongly affected by abandonment. This result shows that grassland species may persist in an abandoned grassland for quite a long time after the cessation of agricultural use, though they disappear from large proportions of the habitat [7]. Our results show that plant groups most affected by cessation of mowing were woody species and invasive species (*Solidago gigantea*) with more than four-fold increase in mean cover. One of the major drivers of the biodiversity in grasslands is the response of dominant species to change in management practises [39]. Previous studies reported that lowland grasslands are particularly susceptible to invasion by neophytes [10,40] and expansion of woody species after the cessation of agricultural use [7], which is also supported by our findings.

We showed that the number of plant species per plot and the average species diversity significantly decreased after 10 years of abandonment. The observed decrease in species richness and plant diversity is consistent with previous studies investigating successional changes over similar durations of abandonment [6,41]. Our results also suggest that extensive management in the form of late mowing once every two years does not fully prevent *Solidago gigantea* encroachment into lowland grassland systems, but does considerably hamper its growth and reproduction. Moderate expansion of *Solidago species* into extensively mown grasslands has also been reported [42]. Periodic management by occasional cutting (once a few years), which is considered a pro-conservation alternative to total abandonment [6,43] might be the optimal management practice in lowland grasslands. It is reasonable to presume that the expansion rate of invasive species will increase immediately after cessation of mowing in extensively used grasslands, therefore, agri-environmental policies should be aware of this risk.

### Relative importance of soil properties and management

The amount of variation in species composition explained by all variables included in the model was quite small when compared with the results of previous studies that included a broad array of environmental, land use and topography-related factors across large gradients of semi-natural vegetation types in the landscape [13,44]. It was rather expected that the amount of variation explained in species composition would be smaller when considering a narrow environmental gradient. The two groups of variables (soil properties and management) included in the model differed considerably in their ability to explain variation in species data. Decomposition of the explained variation reveals the high importance of soil properties and the relatively small impact of recent abandonment on species composition. Our results support the view that plant species composition in grasslands is largely determined by site-specific abiotic conditions, while management has a smaller impact [13]. It is concluded that in extensively used grasslands, the abiotic site conditions are of utmost importance. The impact of abandonment on species composition might, however, become more important in the longer-term with the progressing succession of vegetation. Among soil variables, Ca and pH were the most important predictors of plant species composition. Similarly, these two factors are the most important determinants of species composition in *Molinion* meadows in Slovenia [45]. Soil pH and Ca concentration are among the most important factors controlling plant species composition, as both affect nutrient availability and uptake. A notable finding is that, after partialling out the effects of all soil variables, management has a higher explanatory power than does any soil variable alone with the exceptions of pH and Ca. This shows that the impact of soil parameters on species composition has an interactive rather than an individualistic nature. The underlying explanation is that the different species use nutrients in many ways, particularly in diverse ecosystems where different species specialise in specific resources [46].

Contrary to our hypothesis, the shared effect of soil properties and management on species composition was only a small fraction of explained variance [43,47]. This suggests that soil and management factors explain specific but different aspects of species composition variation [48]. On the other hand, species distribution patterns are governed to a large extent by stochastic processes such as plant-plant interactions, especially at fine spatial scales [49]. Considering the complexity of ecological processes, finding mutual relationships between soil properties and management practises effects with the variation partitioning method is challenging or even practically unfeasible, at least at a fine spatial scale.

### Impact of management on soil properties

The results show that management can markedly influence soil properties. An important finding from this study was that mown sites were more nutrient-rich compared to unmown sites. Note, however, that both sites have a low nutrient status, which is comparable to that reported from *Molinion* meadows in Slovenia [45]. As reported previously, decrease in plant-available concentrations of K in the soil is usually observed in grasslands cut for hay [21,50,51]. However, impoverishment in plant-available N and P, as a result of mowing, have also been reported in several studies [22,23]. Considering the results of this study and others, nutrient depletion patterns in grasslands might therefore be difficult to generalize over different soils, vegetation types and management regimes [20]. At our site, the low intensity and late timing of mowing reduce the export of nutrients from the ecosystem to negligible levels. This suggests that low-intensity and late mowing help to balance soil nutritional status in grasslands. Our results indicate that low-intensity mowing enhances the accumulation of SOM, which is in line with previously reported observations from grasslands [52–54]. As mown meadows are cut every second year, a large proportion of above-ground biomass is available for decomposition. It has been evidenced from earlier studies, however, that root biomass and root exudation are also an important input of organic matter [54–56]. Higher levels of plant-available nutrients in mown meadows can be related to the differences in SOM quality between mown and unmown sites. Although we have not measured the quality of SOM, there is abundant evidence from previous studies that SOM is more susceptible to decomposition in mown grasslands than in unmown grasslands owing to larger supplies of easily degradable carbon and increased microbial activity under managed sites [57,58].

Nutrient impoverishment accompanying secondary succession in grasslands has been poorly demonstrated to date, except in studies investigating the abandonment of previously pastured grasslands [41,59,60]. Land-use induced changes in the content of nutrients are generally very slow processes [61], although a period of ten years is considered long enough to permit detectable alterations in nutrient availability [2], which also supported by our results. Results of studies showing minor or no changes in soil properties after cessation of mowing can be attributed to the low successional rates [19,62], while short-term experiments may not detect land-use induced changes in soil properties at all [17,18]. Our results show that the abundance of woody species is negatively correlated with the content of SOM as well as with other soil parameters. Recent studies have shown that litter quality tends to decline with subsequent phases of succession of grasslands [63–65], and that the physico-chemical characteristics of plants constitute an important factor regulating the dynamics of SOM and the rates of nutrient release [66]. Quested et al. [65] found that decomposition rates of plant residuals is significantly higher in mowed grasslands than in abandoned ones. It has been previously reported that colonisation of grasslands by woody plants leads to losses of labile soil organic carbon [67,68]. Moreover, abandonment of agricultural management favours the growth of nutrient-conservative species such as *Solidago gigantea*. This species is thought to have higher nutrient uptake rates but less degradable litter than native plants owing to the higher C:N ratio in its plant biomass [69]. The results of our Spearman correlation test, indicate a slight trend towards invasion-induced changes in soil properties. Considering the low content of SOM (<5%) under *Solidago*-dominated stands reported by Szymura and Szymura [70], we believe the impact of this species on soil properties might become more important in longer-term successional trajectories. *Solidago* species have been found to change soil properties in several divergent ways [69,71,72]. Different effects of this species on soil properties may depend on the initial species composition, soil type and climatic conditions, which subsequently determine the direction and magnitude of ecosystem-level impacts [69,73,74]. Considering our limited knowledge on the effect of *Solidago* species on soil properties in grasslands, this matter is still far from being explored, and invasion-induced changes in soil require further examination.

Last but not least, our study revealed higher moisture content in soils from mown meadows compared to unmown sites [75]. We found no differences in the overall humidity of soil as expressed by EIVm, even though successional changes in vegetation composition and structure can modify evaporative water losses via intensified community transpiration rates [76,77]. Higher moisture content in mown meadows can therefore be related to the higher SOM content in topsoil, which has a beneficial effect on water holding capacity [75,78].

## Conclusions

This study shows that management can markedly influence both vegetation parameters and soil properties. Our findings also show that the direct effect of soil properties is likely to be much more important for species composition patterns than the direct effect of management. Changes in soil properties are attributable to abandonment and successional changes in vegetation coverage. Note that it is strongly implied that the purpose of this study was to generate conclusions about diversity and nutrient-conserving maintenance practices for semi-natural grasslands. It appears that low-intensity and late time of mowing is suitable grassland management practice to ensure sustainability of the grassland ecological system, while cessation of mowing not only lead to reduced plant species richness and diversity, but also to reduced nutrient levels in grassland soils, at least during the early stages of succession.

## Supporting information

S1 Table Frequency (%) and mean cover (%) of species in mown and unmown sites. Species are sorted alphabetically within distinguished functional species groups.
(DOCX)

## References

[1] Isselstein J, Jeangros B, Pavlu V. Agronomic aspects of biodiversity targeted management of temperate grasslands in Europe–a review. Agron Res 2005;3:139–151.

[2] Bakker JP. Nature management by grazing and cutting Kluwer Academic Publishers, Dordrecht, Boston, London; 1989.

[3] Blackstock TH, Rimes CA, Stevens DP, Jefferson RG, Robertson HJ, Mackintosh J, et al The extent of semi-natural grassland communities in lowland England and Wales: a review of conservation surveys 1978–96. Grass Forage Sci 1999;54:1–18. https://doi.org/10.1046/j.1365-2494.1999.00157.x

[4] Fuchs R, Herold M, Verburg PH, Clevers JGPW. A high-resolution and harmonized model approach for reconstructing and analysing historic land changes in Europe. Biogeosciences 2013;10:1543–1559. https://doi.org/10.5194/bg-10-1543-2013

[5] Kącki Z, Michalska-Hejduk D. Assessment of biodiversity in *Molinia* meadows in Kampinoski National Park based on biocenotic indicators. Pol J Environ Stud 2010;19:351–362.

[6] Pavlů L, Pavlů V, Gaisler J, Hejcman M, Mikulka J. Effect of long-term cutting versus abandonment on the vegetation of a mountain hay meadow (*Polygono-Trisetion*) in Central Europe. Flora 2011;206:1020–1029. https://doi.org/10.1016/j.flora.2011.07.008

[7] Falińska K. Plant population processes in the course of forest succession in abandoned meadows. I. Variability and diversity of floristic compositions, and biological mechanisms of species turnover. Acta Soc Bot Pol 1989;58:439–465. https://doi.org/10.5586/asbp.1989.036

[8] Palmer M. Variation in species richness: towards a unification of hypotheses. Folia Geobot Phytotaxon 1994;29:511–530. https://doi.org/10.1007/BF02883148

[9] Rosenthal G. Secondary succession in a fallow central European wet grassland. Flora 2010;205:153–160.

[10] Weber E, Jakobs G. Biological flora of central Europe: *Solidago gigantea* Aiton. Flora 2005;200:109–118. https://doi.org/10.1016/j.flora.2004.09.001

[11] Gaujour E, Amiaud B, Mignolet C, Plantureux S. Factors and processes affecting plant biodiversity in permanent grasslands. A review. Agron Sustainable Dev 2012;32:133–160. https://doi.org/10.1007/s13593-011-0015-3

[12] Lavorel S, Garnier E. Predicting changes in community composition and ecosystem functioning from plant traits: revisiting the Holy Grail. Funct Ecol 2002;16:545–556. https://doi.org/10.1046/j.1365-2435.2002.00664.x

[13] Myklestad Å. Soil, site and management components of variation in species composition of agricultural grasslands in western Norway. Grass Forage Sci 2004;59:136–143. https://doi.org/10.1111/j.1365-2494.2004.00413.x

[14] Wellstein C, Otte A, Waldhardt R. Impact of site and management on the diversity of central European mesic grassland. Agric Ecosyst Environ 2007;122:203–210. https://doi.org/10.1016/j.agee.2006.12.033

[15] Vandvik V, Birks HJB. Partitioning floristic variance in Norwegian upland grasslands into within-site and between-site components: are the patterns determined by environment or by land-use? Plant Ecol 2002;162:233–245. https://doi.org/10.1023/A:1020322205469

[16] Austrheim G, Gunilla E, Olsson A, Grøntvedt E. Land-use impact on plant communities in semi-natural sub-alpine grasslands of Budalen, central Norway. Biol Conserv 87:369–379; 1999 https://doi.org/10.1016/S0006-3207(98)00071-8

[17] Ilmarinen K, Mikola J. Soil feedback does not explain mowing effects on vegetation structure in a semi-natural grassland. Acta Oecol 2009;35:838–848. https://doi.org/10.1016/j.actao.2009.08.008

[18] Ilmarinen K, Mikola J, Nissinen K, Vestberg M. Role of Soil Organisms in the Maintenance of Species‐Rich Seminatural Grasslands through Mowing. Restor Ecol 2009;17:78–88. https://doi.org/10.1111/j.1526-100X.2007.00341.x

[19] Sørensen LI, Kytöviita MM, Olofsson J, Mikola J. Soil feedback on plant growth in a sub-arctic grassland as a result of repeated defoliation. Soil Biol Biochem 2008;40:2891–2897. https://doi.org/10.1016/j.soilbio.2008.08.009

[20] Rumpel C, Crème A, Ngo PT, Velásquez G, Mora ML, Chabbi A. The impact of grassland management on biogeochemical cycles involving carbon, nitrogen and phosphorus. J Soil Sci Plant Nutr 2015;15:353–371. http://doi.org/10.4067/S0718-95162015005000034

[21] Pavlů L, Pavlů V, Gaisler J, Hejcman M. Relationship between soil and biomass chemical properties, herbage yield and sward height in cut and unmanaged mountain hay meadow (*Polygono-Trisetion*). Flora 2013;208:599–608. https://doi.org/10.1016/j.flora.2013.09.003

[22] Kotas P, Choma M, Šantrůčková H, Lepš J, Tříska J, Kaštovská E. Linking above-and belowground responses to 16 years of fertilization, mowing, and removal of the dominant species in a temperate grassland. Ecosyst 2017;20:354–367. https://doi.org/10.1007/s10021-016-0031-x

[23] Oelmann Y, Broll G, Hölzel N, Kleinebecker T, Vogel A, Schwartzed P. Nutrient impoverishment and limitation of productivity after 20 years of conservation management in wet grasslands of north-western Germany. Biol Conserv 2009;142:2941–2948. https://doi.org/10.1016/j.biocon.2009.07.021

[24] Hejcman M, Češková M, Pavlů V. Control of *Molinia caerulea* by cutting management on sub-alpine grassland. Flora 2010;205:577–582. https://doi.org/10.1016/j.flora.2010.04.019

[25] Borcard D, Legendre P, Drapeau P. Partialling out the spatial component of ecological variation. Ecol 1992;73:1045–1055. https://doi.org/10.2307/1940179

[26] Swacha G, Botta-Dukát Z, Kącki Z, Pruchniewicz D, Żołnierz L. A performance comparison of sampling methods in the assessment of species composition patterns and environment-vegetation relationships in species-rich grasslands. Acta Soc Bot Pol 2017;86:3561 https://doi.org/10.5586/asbp.3561

[27] Walker LR, Wardle DA, Bardgett RD, Clarkson BD. The use of chronosequences in studies of ecological succession and soil development. J Ecol 2010;98:725–736. https://doi.org/10.1111/j.1365-2745.2010.01664.x

[28] Pawlak W. Atlas of Lower and Opole Silesia. Wroclaw University, Wroclaw; 2008.

[29] Goslee SC. Behaviour of vegetation sampling methods in the presence of spatial autocorrelation. Plant Ecol 2006;187:203–212. https://doi.org/10.1007/s11258-005-3495-x

[30] Westhoff V, van der Maarel E. The Braun-Blanquet approach, in: Whittaker R.H. (ed.) Classification of plant communities. W. Junk, The Hague, pp. 289–399; 1978.

[31] Allen SE. Chemical Analysis of Ecological Materials, second ed. completely revised Blackwell Scientific Publications, Oxford, London, Edinburgh; 1989.

[32] Radojević M, Bashkin VN. Practical Environmental Analysis. Royal Society of Chemistry, Cambridge; 2006.

[33] Ellenberg H, Weber HE, Düll R, Wirth V, Werner W, Paulißen D. Zeigerwerte von Pflanzen in Mitteleuropa. Scr Geobot 1992;18:3–258.

[34] Schaffers AP, Sýkora KV. Reliability of Ellenberg indicator values for moisture, nitrogen and soil reaction: a comparison with field measurements. J Veg Sci 2000;11:225–244. http://doi.org/10.2307/3236802

[35] Fox J, Monette G. Generalized collinearity diagnostics. JASA 1992;87:178–183. https://doi.org/10.2307/2290467

[36] Legendre P, Gallagher ED. Ecologically meaningful transformations for ordination of species data. Oecologia 2001;129:271–280. https://doi.org/10.1007/s004420100716 28547606 10.1007/s004420100716

[37] Legendre P, Legendre L. Numerical Ecology, second English ed. Elsevier Science, Amsterdam; 1998.

[38] ter Braak CJF, Šmilauer P. Canoco Reference Manual and User's Guide: Software for Ordination (version 5.0). Microcomputer Power, Ithaca, New York, USA; 2012.

[39] Lepš J. Scale‐and time‐dependent effects of fertilization, mowing and dominant removal on a grassland community during a 15‐year experiment. J Appl Ecol 2014;51:978–987.

[40] Pyšek P, Jarošík V, Kučera T. Patterns of invasion in temperate nature reserves. Biol Conserv 2002;104:13–24. https://doi.org/10.1016/S0006-3207(01)00150-1

[41] Hansson M, Fogelfors H. Management of a semi‐natural grassland; results from a 15‐year‐old experiment in southern Sweden. J Veg Sci 2000;11:31–38. https://doi.org/10.2307/3236772

[42] Kőrösi Á, Szentirmai I, Batáry P, Kövér S, Örvössy N, Peregovits L. Effects of timing and frequency of mowing on the threatened scarce large blue butterfly–A fine-scale experiment. Agric Ecosyst Environ 2014;196: 24–33. https://doi.org/10.1016/j.agee.2014.06.019

[43] Rudmann-Maurer K, Weyand A, Fischer M, Stöcklin J. The role of landuse and natural determinants for grassland vegetation composition in the Swiss Alps. Basic Appl Ecol 2008;9:494–503. https://doi.org/10.1016/j.baae.2007.08.005

[44] Klimek S, Hofmann M, Isselstein J. Plant species richness and composition in managed grasslands: the relative importance of field management and environmental factors. Biol Conserv 2007;134:559–570. https://doi.org/10.1016/j.biocon.2006.09.007

[45] Zelnik I, Čarni A. Wet meadows of the alliance *Molinion* and their environmental gradients in Slovenia. Biologia 2008;63:187–196. https://doi.org/10.2478/s11756-008-0042-y

[46] Lundholm JT. Plant species diversity and environmental heterogeneity: spatial scale and competing hypotheses. J Veg Sci 2009;20:377–391. https://doi.org/10.1111/j.1654-1103.2009.05577.x

[47] Pinke G, Karácsony P, Czúcz B, Botta‐Dukát Z, Lengyel A. The influence of environment, management and site context on species composition of summer arable weed vegetation in Hungary. Appl Veg Sci 2012;15:136–144. https://doi.org/10.1111/j.1654-109X.2011.01158.x

[48] Ribeiro S, Fernandes JP, Espírito-Santo MD. Diversity and floristic patterns of mediterranean grasslands: the relative influence of environmental and land management factors. Biodivers Conserv 2014;23:2903–2921. https://doi.org/10.1007/s10531-014-0754-y

[49] Richard M, Bernhardt T, Bell G. Environmental heterogeneity and the spatial structure of fern species diversity in one hectare of old‐growth forest. Ecography 2000;23:231–245. https://doi.org/10.1111/j.1600-0587.2000.tb00279.x

[50] Mládková P, Mládek J, Hejduk S, Hejcman M, Cruz P, Jouany C, et al High‐nature‐value grasslands have the capacity to cope with nutrient impoverishment induced by mowing and livestock grazing. J Appl Ecol 2015;52:1073–1081. https://doi.org/10.1111/1365-2664.12464

[51] Pruchniewicz D, Żołnierz L. The influence of environmental factors and management methods on the vegetation of mesic grasslands in a central European mountain range. Flora 2014;209:687–692. https://doi.org/10.1016/j.flora.2014.09.001

[52] Guidi C, Vesterdal L, Gianelle D, Rodeghiero M. Changes in soil organic carbon and nitrogen following forest expansion on grassland in the Southern Alps. For Ecol Manage 2014;328:103–116. https://doi.org/10.1016/j.foreco.2014.05.025

[53] Li J, Zhang Q, Li Y, Liu Y, Xu J, Di H. Effects of long-term mowing on the fractions and chemical composition of soil organic matter in a semiarid grassland. Biogeosciences 2017;14:2685 https://doi.org/10.5194/bg-14-2685-2017

[54] Ziter C, MacDougall AS. Nutrients and defoliation increase soil carbon inputs in grassland. Ecol 2013;94:106–116. 10.1890/11-2070.1 23600245

[55] Cong WF, Ruijven J, Mommer L, De Deyn GB, Berendse F, Hoffland E. Plant species richness promotes soil carbon and nitrogen stocks in grasslands without legumes. J Ecol 2014;102:1163–1170. https://doi.org/10.1111/1365-2745.12280

[56] Freschet GT, Cornwell WK, Wardle DA, Elumeeva TG, Liu W, Jackson BG, et al Linking litter decomposition of above‐and below‐ground organs to plant–soil feedbacks worldwide. J Ecol 2013;101:943–952. https://doi.org/10.1111/1365-2745.12092

[57] Bardgett RD, Wardle DA, Yeates GW. Linking aboveground and below-ground interactions: how plant responses to foliar herbivory influence soil organisms. Soil Biol Biochem 1998;30:1867–1878. https://doi.org/10.1016/S0038-0717(98)00069-8

[58] Uhlířová E, Šimek M, Šantrůčková H. Microbial transformation of organic matter in soils of montane grasslands under different management. Appl Soil Ecol 2005;28:225–235. https://doi.org/10.1016/j.apsoil.2004.08.002

[59] Marriott CA, Fisher JM, Hood K, Pakeman RJ. Impacts of extensive grazing and abandonment on grassland soils and productivity. Agric Ecosyst Environ 2010;139:476–482. https://doi.org/10.1016/j.agee.2010.09.005

[60] Peco B, Navarro E, Carmona CP, Medina NG, Marques MJ. Effects of grazing abandonment on soil multifunctionality: The role of plant functional traits. Agric Ecosyst Environ 2017;249:215–225. https://doi.org/10.1016/j.agee.2017.08.013

[61] Pavlů L, Gaisler J, Hejcman M, Pavlů V. What is the effect of long-term mulching and traditional cutting regimes on soil and biomass chemical properties, species richness and herbage production in *Dactylis glomerata* grassland? Agric Ecosyst Environ 2016;217:13–21. https://doi.org/10.1016/j.agee.2015.10.026

[62] Köhler B, Ryser P, Güsewell S, Gigon A. Nutrient availability and limitation in traditionally mown and in abandoned limestone grasslands: a bioassay experiment. Plant Soil 2001;230:323–332. https://doi.org/10.1023/A:1010335825818

[63] Gamper SM, Tasser E, Tappeiner U. Short-time effects of land-use changes on O-horizon in subalpine grasslands. Plant Soil 2007;299:101–115. https://doi.org/10.1007/s11104-007-9366-6

[64] Maharning AR, Mills AA, Adl SM. Soil community changes during secondary succession to naturalized grasslands. Appl Soil Ecol 2009;41:137–147. https://doi.org/10.1016/j.apsoil.2008.11.003

[65] Quested H, Eriksson O, Fortunel C, Garnier E. Plant traits relate to whole‐community litter quality and decomposition following land use change. Funct Ecol 2007;21:1016–1026. https://doi.org/10.1111/j.1365-2435.2007.01324.x

[66] Cotrufo MF, Wallenstein MD, Boot CM, Denef K, Paul E. The Microbial Efficiency‐Matrix Stabilization (MEMS) framework integrates plant litter decomposition with soil organic matter stabilization: do labile plant inputs form stable soil organic matter? Glob Chang Biol 2013;19:988–995. https://doi.org/10.1111/gcb.12113 23504877 10.1111/gcb.12113

[67] Jackson RB, Banner JL, Jobbágy EG, Pockman WT, Wall DH. Ecosystem carbon loss with woody plant invasion of grasslands. Nature 2002;418:623–626. doi: 10.1038/nature00910 12167857 10.1038/nature00910

[68] Hiltbrunner D, Zimmermann S, Hagedorn F. Afforestation with Norway spruce on a subalpine pasture alters carbon dynamics but only moderately affects soil carbon storage. Biogeochemistry 2013;115:251–266. https://doi.org/10.1007/s10533-013-9832-6

[69] Scharfy D, Eggenschwiler H, Olde Venterink H, Edwards PJ, Güsewell S. The invasive alien plant species *Solidago gigantea* alters ecosystem properties across habitats with differing fertility. J Veg Sci 2009;20:1072–1085. http://doi.org/10.1111/j.1654-1103.2009.01105.x

[70] Szymura M, Szymura TH. Soil preferences and morphological diversity of goldenrods (*Solidago* L.) from south-western Poland. Acta Soc Bot Pol 2013;82:107–115. https://doi.org/10.5586/asbp.2013.005

[71] Chapuis-Lardy L, Vanderhoeven S, Dassonville N, Koutika LS, Meerts P. Effect of the exotic invasive plant *Solidago gigantea* on soil phosphorus status. Biol Fertil Soils 2006;42:481–489. https://doi.org/10.1007/s00374-005-0039-4

[72] Stefanowicz AM, Stanek M, Nobis M, Zubek S. Few effects of invasive plants Reynoutria japonica, Rudbeckia laciniata and *Solidago gigantea* on soil physical and chemical properties. Sci Total Environ 2017;574:938–946. https://doi.org/10.1016/j.scitotenv.2016.09.120 27665453 10.1016/j.scitotenv.2016.09.120

[73] Ehrenfeld JG. Effects of exotic plant invasions on soil nutrient cycling processes. Ecosystems 2003;6:503–523. https://doi.org/10.1007/s10021-002-0151-3

[74] Dassonville N, Vanderhoeven S, Vanparys V, Hayez M, Gruber W, Meerts P. Impacts of alien invasive plants on soil nutrients are correlated with initial site conditions in NW Europe. Oecologia 2008;157:131–140. https://doi.org/10.1007/s00442-008-1054-6 18491146 10.1007/s00442-008-1054-6

[75] Chmolowska D, Kozak M, Laskowski R. Soil physicochemical properties and floristic composition of two ecosystems differing in plant diversity: fallows and meadows. Plant Soil 2016;402:317–329. https://doi.org/10.1007/s11104-015-2788-7

[76] Nosetto MD, Jobbagy EG, Paruelo JM. Land‐use change and water losses: the case of grassland afforestation across a soil textural gradient in central Argentina. Glob Chang Biol 2005;11:1101–1117. https://doi.org/10.1111/j.1365-2486.2005.00975.x

[77] Gross N, Robson TM, Lavorel S, Albert C, Bagousse‐Pinguet L, Guillemin R. Plant response traits mediate the effects of subalpine grasslands on soil moisture. New Phytol 2008;180:652–662. https://doi.org/10.1111/j.1469-8137.2008.02577.x 18657216 10.1111/j.1469-8137.2008.02577.x

[78] Hudson BD; Soil organic matter and available water capacity. J. Soil Water Conserv 1994;49:189–194.

