# Supplementary material for: The effect of abandonment on vegetation composition and soil properties in Molinion meadows (SW Poland)
Source: PLoS One. 2018 May 17;13(5):e0197363. doi: 10.1371/journal.pone.0197363 (PMC5957338; doi:10.1371/journal.pone.0197363)
Supplement: S1 Table — Species are sorted alphabetically within distinguished functional species groups. (DOCX) [file pone.0197363.s001.docx]

Supporting information S1 Table. Frequency (%) and mean cover (%) of species in mown and unmown sites. Species are sorted alphabetically within distinguished functional plant species groups.

| Species | Functional group | Mown |  | Unmown | |
| --- | --- | --- | --- | --- | --- |
|  |  | Frequency (%) | Mean cover (%) | Frequency (%) | Mean cover (%) |
| *Agrostis canina* | Monocots | 28 | 2.5 | 19 | 1.0 |
| *Agrostis capillaris* | Monocots | 36 | 1.6 | 48 | 2.2 |
| *Agrostis gigantea* | Monocots | 3 | 0.1 | 0 | 0 |
| *Agrostis stolonifera* | Monocots | 6 | 0.1 | 0 | 0 |
| *Allium angulosum* | Monocots | 2 | <0.1 | 0 | 0 |
| *Allium vineale* | Monocots | 1 | <0.1 | 0 | 0 |
| *Alopecurus pratensis* | Monocots | 57 | 3.9 | 50 | 8.0 |
| *Anthoxanthum odoratum* | Monocots | 47 | 1.1 | 33 | 0.7 |
| *Arrhenatherum elatius* | Monocots | 41 | 1.3 | 61 | 2.0 |
| *Avenella flexuosa* | Monocots | 0 | 0 | 1 | 0.2 |
| *Avenula pubescens* | Monocots | 8 | 0.2 | 8 | 0.2 |
| *Briza media* | Monocots | 19 | 0.4 | 21 | 0.4 |
| *Bromopsis inermis* | Monocots | 1 | 0.1 | 1 | <0.1 |
| *Bromus secalinus* | Monocots | 0 | 0 | 1 | <0.1 |
| *Calamagrostis epigejos* | Monocots | 58 | 13.3 | 30 | 5.6 |
| *Carex acuta* | Monocots | 5 | 0.2 | 1 | <0.1 |
| *Carex acutiformis* | Monocots | 46 | 4.4 | 40 | 2.9 |
| *Carex brizoides* | Monocots | 2 | 0.3 | 1 | 0.2 |
| *Carex filiformis* | Monocots | 38 | 1.8 | 10 | 0.2 |
| *Carex flacca* | Monocots | 14 | 0.3 | 8 | 0.2 |
| *Carex flava* | Monocots | 2 | <0.1 | 0 | 0 |
| *Carex hartmanii* | Monocots | 43 | 2.2 | 25 | 1.0 |
| *Carex hirta* | Monocots | 57 | 1.3 | 74 | 2.5 |
| *Carex leporina* | Monocots | 8 | 0.2 | 5 | 0.1 |
| *Carex muricata* | Monocots | 5 | 0.1 | 8 | 0.2 |
| *Carex nigra* | Monocots | 10 | 0.2 | 5 | 0.1 |
| *Carex pallescens* | Monocots | 59 | 1.3 | 29 | 0.6 |
| *Carex panicea* | Monocots | 54 | 3.7 | 43 | 2.2 |
| *Carex pilulifera* | Monocots | 2 | <0.1 | 0 | 0 |
| *Carex praecox* | Monocots | 1 | <0.1 | 0 | 0 |
| *Carex riparia* | Monocots | 2 | <0.1 | 0 | 0 |
| *Carex rostrata* | Monocots | 2 | 0.1 | 0 | 0 |
| *Carex umbrosa* | Monocots | 2 | <0.1 | 0 | 0 |
| *Carex vesicaria* | Monocots | 2 | <0.1 | 1 | <0.1 |
| *Carex vulpina* | Monocots | 11 | 0.4 | 10 | 0.4 |
| *Dactylis glomerata* | Monocots | 45 | 1.0 | 28 | 0.6 |
| *Dactylorhiza majalis* | Monocots | 4 | 0.1 | 3 | 0.1 |
| *Danthonia decumbens* | Monocots | 8 | 0.2 | 9 | 0.2 |
| *Deschampsia caespitosa* | Monocots | 58 | 1.6 | 60 | 1.5 |
| *Elytrigia repens* | Monocots | 2 | <0.1 | 4 | 0.1 |
| *Festuca ovina* | Monocots | 1 | <0.1 | 1 | <0.1 |
| *Festuca rubra* | Monocots | 61 | 5.7 | 66 | 7.5 |
| *Gladiolus imbricatus* | Monocots | 1 | <0.1 | 1 | <0.1 |
| *Holcus lanatus* | Monocots | 72 | 1.8 | 66 | 1.4 |
| *Holcus mollis* | Monocots | 2 | <0.1 | 3 | 0.2 |
| *Iris pseudacorus* | Monocots | 5 | 0.3 | 0 | 0 |
| *Juncus articulatus* | Monocots | 9 | 0.2 | 8 | 0.9 |
| *Juncus bufonius* | Monocots | 1 | <0.1 | 0 | 0 |
| *Juncus compressus* | Monocots | 1 | <0.1 | 0 | 0 |
| *Juncus conglomeratus* | Monocots | 55 | 1.1 | 45 | 0.9 |
| *Juncus effusus* | Monocots | 27 | 0.6 | 18 | 0.5 |
| *Juncus inflexus* | Monocots | 6 | 0.1 | 6 | 0.1 |
| *Luzula campestris* | Monocots | 5 | 0.1 | 8 | 0.2 |
| *Luzula multiflora* | Monocots | 38 | 0.9 | 24 | 0.5 |
| *Molinia caerulea* | Monocots | 73 | 13.1 | 46 | 8.7 |
| *Nardus stricta* | Monocots | 2 | <0.1 | 1 | <0.1 |
| *Neottia ovata* | Monocots | 1 | <0.1 | 3 | 0.1 |
| *Phalaroides arundinacea* | Monocots | 6 | 0.1 | 1 | <0.1 |
| *Phleum pratense* | Monocots | 38 | 1.1 | 28 | 0.6 |
| *Phragmites australis* | Monocots | 3 | 0.4 | 3 | 0.9 |
| *Poa nemoralis* | Monocots | 0 | 0 | 1 | <0.1 |
| *Poa palustris* | Monocots | 9 | 0.2 | 1 | <0.1 |
| *Poa pratensis* | Monocots | 33 | 1.0 | 40 | 1.8 |
| *Poa remota* | Monocots | 1 | <0.1 | 0 | 0 |
| *Poa trivialis* | Monocots | 27 | 0.8 | 16 | 0.4 |
| *Schedonorus arundinaceus* | Monocots | 8 | 0.2 | 8 | 0.3 |
| *Schedonorus pratensis* | Monocots | 18 | 0.4 | 21 | 0.6 |
| *Scirpus sylvaticus* | Monocots | 4 | 0.2 | 10 | 0.9 |
| *Secale cereale* | Monocots | 1 | <0.1 | 1 | <0.1 |
| *Trisetum flavescens* | Monocots | 8 | 0.2 | 0 | 0 |
| *Achillea millefolium* | Dicots | 53 | 1.2 | 53 | 1.1 |
| *Achillea ptarmica* | Dicots | 43 | 0.9 | 28 | 0.9 |
| *Aegopodium podagraria* | Dicots | 2 | <0.1 | 0 | 0 |
| *Agrimonia eupatoria* | Dicots | 25 | 0.8 | 24 | 0.5 |
| *Ajuga reptans* | Dicots | 33 | 0.7 | 33 | 0.7 |
| *Alnus glutinosa* [seedling] | Dicots | 3 | 0.1 | 1 | <0.1 |
| *Anemone nemorosa* | Dicots | 13 | 0.3 | 14 | 0.3 |
| *Angelica sylvestris* | Dicots | 7 | 0.1 | 1 | <0.1 |
| *Argentina anserina* | Dicots | 17 | 0.5 | 10 | 0.2 |
| *Artemisia vulgaris* | Dicots | 0 | 0 | 3 | 0.1 |
| *Bellis perennis* | Dicots | 1 | <0.1 | 0 | 0 |
| *Betula pendula* [seedling] | Dicots | 13 | 0.3 | 5 | 0.1 |
| *Bidens frondosus* | Dicots | 3 | 0.1 | 0 | 0 |
| *Bistorta officinalis* | Dicots | 3 | 0.2 | 6 | 0.3 |
| *Calluna vulgaris* | Dicots | 0 | 0 | 1 | <0.1 |
| *Caltha palustris* | Dicots | 1 | <0.1 | 0 | 0 |
| *Calystegia sepium* | Dicots | 2 | <0.1 | 3 | 0.1 |
| *Campanula glomerata* | Dicots | 1 | <0.1 | 1 | <0.1 |
| *Campanula patula* | Dicots | 32 | 0.6 | 26 | 0.5 |
| *Cardamine pratensis* | Dicots | 22 | 0.4 | 5 | 0.1 |
| *Carduus acanthoides* | Dicots | 2 | <0.1 | 1 | <0.1 |
| *Carpinus betulus [seedling]* | Dicots | 2 | <0.1 | 1 | <0.1 |
| *Centaurea jacea* | Dicots | 65 | 1.5 | 50 | 1.3 |
| *Centaurium erythraea* | Dicots | 5 | 0.1 | 1 | <0.1 |
| *Cerastium fontanum subsp. vulgare* | Dicots | 31 | 0.6 | 9 | 0.2 |
| *Cirsium arvense* | Dicots | 54 | 1.4 | 71 | 1.7 |
| *Cirsium canum* | Dicots | 47 | 3.4 | 20 | 2.4 |
| *Cirsium oleraceum* | Dicots | 2 | 0.1 | 1 | <1 |
| *Cirsium palustre* | Dicots | 36 | 0.7 | 23 | 0.5 |
| *Clematis vitalba* | Dicots | 0 | 0 | 3 | 0.1 |
| *Cornus sanguinea* [seedling] | Dicots | 1 | <0.1 | 0 | 0 |
| *Corylus avellana* [seedling] | Dicots | 0 | 0 | 3 | 0.1 |
| *Crataegus monogyna* [seedling] | Dicots | 14 | 0.3 | 11 | 0.2 |
| *Crepis paludosa* | Dicots | 1 | <0.1 | 0 | 0 |
| *Daucus carota* | Dicots | 7 | 0.2 | 8 | 0.2 |
| *Dianthus deltoides* | Dicots | 10 | 0.2 | 4 | 0.1 |
| *Dryopteris carthusiana* | Dicots | 4 | 0.1 | 3 | 0.1 |
| *Epilobium angustifolium* | Dicots | 1 | <0.1 | 1 | <0.1 |
| *Epilobium hirsutum* | Dicots | 0 | 0 | 1 | <0.1 |
| *Epilobium palustre* | Dicots | 6 | 0.1 | 4 | 0.1 |
| *Epilobium parviflorum* | Dicots | 1 | <0.1 | 3 | 0.1 |
| *Equisetum arvense* | Dicots | 11 | 0.2 | 21 | 0.4 |
| *Equisetum palustre* | Dicots | 5 | 0.1 | 8 | 0.2 |
| *Equisetum pratense* | Dicots | 7 | 0.1 | 10 | 0.2 |
| *Euonymus europaea* | Dicots | 2 | <0.1 | 0 | 0 |
| *Eupatorium cannabinum* | Dicots | 0 | 0 | 6 | 0.1 |
| *Euphorbia cyparissias* | Dicots | 1 | <0.1 | 6 | 0.1 |
| *Fallopia dumetorum* | Dicots | 3 | 0.1 | 0 | 0 |
| *Filipendula ulmaria* | Dicots | 37 | 5.2 | 29 | 4.5 |
| *Filipendula vulgaris* | Dicots | 45 | 1.1 | 45 | 1.0 |
| *Fragaria vesca* | Dicots | 5 | 0.4 | 3 | 0.2 |
| *Fragaria viridis* | Dicots | 8 | 0.5 | 3 | 0.1 |
| *Frangula alnus* [seedling] | Dicots | 43 | 1.2 | 30 | 0.6 |
| *Fraxinus excelsior* [seedling] | Dicots | 1 | <0.1 | 0 | 0 |
| *Galeopsis tetrahit* | Dicots | 18 | 0.4 | 43 | 1.5 |
| *Galium aparine* | Dicots | 8 | 0.2 | 18 | 0.4 |
| *Galium boreale* | Dicots | 63 | 7.8 | 63 | 4.4 |
| *Galium mollugo* | Dicots | 8 | 0.2 | 4 | 0.1 |
| *Galium palustre* | Dicots | 34 | 0.7 | 20 | 0.4 |
| *Galium uliginosum* | Dicots | 20 | 0.4 | 16 | 0.3 |
| *Galium verum* | Dicots | 36 | 1.2 | 35 | 1.2 |
| *Genista tinctoria* | Dicots | 4 | 0.1 | 4 | 0.1 |
| *Gentiana pneumonanthe* | Dicots | 3 | 0.1 | 1 | <0.1 |
| *Geranium palustre* | Dicots | 4 | 0.1 | 6 | 0.7 |
| *Geum rivale* | Dicots | 8 | 0.3 | 3 | 0.1 |
| *Glechoma hederacea* | Dicots | 18 | 0.4 | 24 | 0.9 |
| *Heracleum sphondylium* | Dicots | 0 | 0 | 3 | 0.1 |
| *Hieracium umbellatum* | Dicots | 8 | 0.2 | 14 | 0.3 |
| *Humulus lupulus* | Dicots | 1 | <0.1 | 0 | 0 |
| *Hypericum maculatum* | Dicots | 9 | 0.2 | 9 | 0.3 |
| *Hypericum perforatum* | Dicots | 13 | 0.6 | 25 | 0.5 |
| *Hypochoeris radicata* | Dicots | 2 | <0.1 | 1 | <0.1 |
| *Impatiens noli-tangere* | Dicots | 1 | <0.1 | 0 | 0 |
| *Impatiens parviflora* | Dicots | 2 | <0.1 | 3 | 0.2 |
| *Inula salicina* | Dicots | 11 | 0.9 | 8 | 0.9 |
| *Jacobaea vulgaris* | Dicots | 1 | <0.1 | 1 | <0.1 |
| *Knautia arvensis* | Dicots | 2 | <0.1 | 4 | 0.1 |
| *Lathyrus palustris* | Dicots | 2 | <0.1 | 0 | 0 |
| *Lathyrus pratensis* | Dicots | 71 | 1.5 | 61 | 1.3 |
| *Leontodon hispidus* | Dicots | 2 | <0.1 | 0 | 0 |
| *Leucanthemum vulgare* | Dicots | 14 | 0.3 | 10 | 0.2 |
| *Lotus corniculatus* | Dicots | 11 | 0.2 | 3 | 0.1 |
| *Lotus pedunculatus* | Dicots | 34 | 0.7 | 14 | 0.3 |
| *Lycopus europaeus* | Dicots | 19 | 0.5 | 6 | 0.1 |
| *Lysimachia nummularia* | Dicots | 38 | 1.6 | 38 | 0.9 |
| *Lysimachia vulgaris* | Dicots | 51 | 2.0 | 49 | 1.8 |
| *Lythrum salicaria* | Dicots | 46 | 1.3 | 30 | 1.1 |
| *Melampyrum pratense* | Dicots | 5 | 0.1 | 5 | 0.1 |
| *Mentha aquatica* | Dicots | 1 | <0.1 | 3 | 0.1 |
| *Mentha arvensis* | Dicots | 38 | 1.6 | 24 | 0.7 |
| *Moehringia trinervia* | Dicots | 0 | 0 | 1 | <0.1 |
| *Myosotis scorpioides* | Dicots | 1 | <0.1 | 1 | <0.1 |
| *Ononis spinosa subsp. hircina* | Dicots | 2 | <0.1 | 0 | 0 |
| *Ophioglossum vulgatum* | Dicots | 24 | 0.5 | 20 | 0.4 |
| *Persicaria amphibia* | Dicots | 2 | <0.1 | 0 | 0 |
| *Persicaria hydropiper* | Dicots | 1 | <0.1 | 0 | 0 |
| *Peucedanum cervaria* | Dicots | 3 | 0.1 | 6 | 0.3 |
| *Peucedanum oreoselinum* | Dicots | 0 | 0 | 8 | 0.8 |
| *Peucedanum palustre* | Dicots | 2 | <0.1 | 3 | 0.1 |
| *Pilosella officinarum* | Dicots | 0 | 0 | 1 | 0.2 |
| *Pimpinella major* | Dicots | 0 | 0 | 1 | <0.1 |
| *Pimpinella saxifraga* | Dicots | 7 | 0.1 | 6 | 0.1 |
| *Pinus sylvestris* [seedling] | Dicots | 0 | 0 | 1 | <0.1 |
| *Plantago lanceolata* | Dicots | 17 | 0.7 | 16 | 0.3 |
| *Plantago major* | Dicots | 3 | 0.1 | 1 | <0.1 |
| *Polygala vulgaris* | Dicots | 0 | 0 | 1 | <0.1 |
| *Populus tremula* [seedling] | Dicots | 9 | 0.2 | 8 | 0.2 |
| *Potentilla alba* | Dicots | 2 | 0.1 | 9 | 0.4 |
| *Potentilla erecta* | Dicots | 45 | 1.1 | 20 | 0.5 |
| *Potentilla reptans* | Dicots | 60 | 3.6 | 63 | 1.9 |
| *Prunella vulgaris* | Dicots | 6 | 0.1 | 1 | <0.1 |
| *Prunus serotina* [seedling] | Dicots | 1 | <0.1 | 5 | 0.1 |
| *Prunus spinosa* [seedling] | Dicots | 32 | 1.0 | 29 | 0.6 |
| *Pyrus communis* | Dicots | 3 | 0.1 | 8 | 0.2 |
| *Quercus petraea* [seedling] | Dicots | 1 | <0.1 | 0 | 0 |
| *Quercus robur* [seedling] | Dicots | 20 | 0.4 | 20 | 0.4 |
| *Quercus rubra* [seedling] | Dicots | 0 | 0 | 1 | <0.1 |
| *Ranunculus acris* | Dicots | 70 | 1.4 | 53 | 1.1 |
| *Ranunculus auricomus* | Dicots | 53 | 1.1 | 43 | 0.9 |
| *Ranunculus flammula* | Dicots | 22 | 0.6 | 5 | 0.2 |
| *Ranunculus polyanthemos* | Dicots | 5 | 0.1 | 4 | 0.1 |
| *Ranunculus repens* | Dicots | 62 | 4.0 | 44 | 1.2 |
| *Rosa canina* [seedling] | Dicots | 18 | 0.4 | 15 | 0.3 |
| *Rosa gallica* | Dicots | 1 | <0.1 | 4 | 0.5 |
| *Rubus caesius* | Dicots | 7 | 0.9 | 5 | 0.3 |
| *Rubus plicatus* [seedling] | Dicots | 9 | 0.2 | 5 | 1.3 |
| *Rumex acetosa* | Dicots | 75 | 1.5 | 70 | 1.4 |
| *Rumex acetosella* | Dicots | 0 | 0 | 1 | <0.1 |
| *Rumex crispus* | Dicots | 1 | <0.1 | 0 | 0 |
| *Rumex obtusifolius* | Dicots | 1 | <0.1 | 0 | 0 |
| *Salix aurita* [seedling] | Dicots | 0 | 0 | 1 | <0.1 |
| *Salix cinerea* [seedling] | Dicots | 10 | 0.5 | 8 | 1.2 |
| *Salix repens* subsp*. rosmarinifolia* [seedling] | Dicots | 0 | 0 | 3 | 0.5 |
| *Sanguisorba officinalis* | Dicots | 84 | 5.4 | 69 | 2.9 |
| *Scorzonera humilis* | Dicots | 2 | <0.1 | 6 | 0.1 |
| *Scorzoneroides autumnalis* | Dicots | 3 | 0.1 | 1 | <0.1 |
| *Scrophularia nodosa* | Dicots | 3 | 0.1 | 13 | 0.3 |
| *Scutellaria galericulata* | Dicots | 1 | <0.1 | 3 | 0.1 |
| *Scutellaria hastifolia* | Dicots | 3 | 0.1 | 1 | <0.1 |
| *Selinum carvifolia* | Dicots | 81 | 2.7 | 65 | 2.0 |
| *Selinum dubium* | Dicots | 40 | 1.6 | 23 | 0.7 |
| *Serratula tinctoria* | Dicots | 32 | 0.8 | 46 | 0.9 |
| *Silaum silaus* | Dicots | 66 | 1.7 | 65 | 1.6 |
| *Silene flos-cuculi* | Dicots | 73 | 1.6 | 63 | 1.3 |
| *Silene latifolia* | Dicots | 1 | <0.1 | 0 | 0 |
| *Silene viscaria* | Dicots | 0 | 0 | 3 | 0.1 |
| *Solidago canadensis* | Dicots | 1 | <0.1 | 3 | 0.1 |
| *Sonchus arvensis* | Dicots | 1 | <0.1 | 0 | 0 |
| *Sorbus aucuparia* | Dicots | 1 | <0.1 | 1 | <0.1 |
| *Stachys officinalis* | Dicots | 74 | 5.1 | 61 | 3.4 |
| *Stachys palustris* | Dicots | 7 | 0.2 | 0 | 0 |
| *Stellaria graminea* | Dicots | 50 | 1.0 | 18 | 0.4 |
| *Stellaria media* | Dicots | 0 | 0 | 1 | <0.1 |
| *Stellaria palustris* | Dicots | 15 | 1.3 | 0 | 0 |
| *Succisa pratensis* | Dicots | 24 | 0.5 | 14 | 0.3 |
| *Symphytum officinale* | Dicots | 3 | 0.1 | 0 | 0 |
| *Tanacetum vulgare* | Dicots | 1 | <0.1 | 3 | 0.1 |
| *Taraxacum sect. Taraxacum* | Dicots | 11 | 0.2 | 6 | 0.1 |
| *Thymus pulegioides* | Dicots | 0 | 0 | 4 | 0.1 |
| *Tilia cordata* [seedling] | Dicots | 2 | <0.1 | 0 | 0 |
| *Torilis japonica* | Dicots | 2 | <0.1 | 3 | 0.1 |
| *Tragopogon pratensis* | Dicots | 1 | <0.1 | 1 | <0.1 |
| *Trifolium medium* | Dicots | 0 | 0 | 1 | <0.1 |
| *Trifolium pratense* | Dicots | 6 | 0.1 | 1 | <0.1 |
| *Trifolium repens* | Dicots | 3 | 0.1 | 0 | 0 |
| *Urtica dioica* | Dicots | 6 | 0.1 | 8 | 0.2 |
| *Valeriana officinalis* | Dicots | 0 | 0 | 1 | <0.1 |
| *Veronica arvensis* | Dicots | 1 | <0.1 | 0 | 0 |
| *Veronica chamaedrys* | Dicots | 36 | 1.1 | 35 | 0.9 |
| *Veronica longifolia* | Dicots | 1 | <0.1 | 0 | 0 |
| *Veronica officinalis* | Dicots | 0 | 0 | 1 | <0.1 |
| *Veronica scutellata* | Dicots | 3 | 0.1 | 0 | 0 |
| *Vicia cracca* | Dicots | 33 | 0.7 | 41 | 0.8 |
| *Vicia sepium* | Dicots | 0 | 0 | 6 | 0.1 |
| *Vicia tetrasperma* | Dicots | 0 | 0 | 1 | <0.1 |
| *Viola canina* | Dicots | 13 | 0.3 | 21 | 0.4 |
| *Viola reichenbachiana* | Dicots | 0 | 0 | 1 | <0.1 |
| *Alnus glutinosa* | Trees and shrubs >0.5 m | 0 | 0 | 1 | <0.1 |
| *Betula pendula* | Trees and shrubs >0.5 m | 5 | 0.2 | 16 | 2.4 |
| *Carpinus betulus* | Trees and shrubs >0.5 m | 0 | 0 | 3 | 0.1 |
| *Cornus sanguinea* | Trees and shrubs >0.5 m | 1 | <0.1 | 0 | 0 |
| *Crataegus monogyna* | Trees and shrubs >0.5 m | 2 | <0.1 | 8 | 0.6 |
| *Frangula alnus* | Trees and shrubs >0.5 m | 35 | 1.1 | 26 | 3.2 |
| *Malus sylvestris* | Trees and shrubs >0.5 m | 0 | 0 | 1 | <0.1 |
| *Pinus sylvestris* | Trees and shrubs >0.5 m | 0 | 0 | 5 | 0.6 |
| *Populus tremula* | Trees and shrubs >0.5 m | 4 | 0.1 | 8 | 0.3 |
| *Prunus cerasus* | Trees and shrubs >0.5 m | 0 | 0 | 1 | 0.5 |
| *Prunus serotina* | Trees and shrubs >0.5 m | 2 | <0.1 | 14 | 1.6 |
| *Prunus spinosa* | Trees and shrubs >0.5 m | 12 | 0.3 | 24 | 1.1 |
| *Pyrus communis* | Trees and shrubs >0.5 m | 3 | 0.1 | 11 | 0.6 |
| *Quercus robur* | Trees and shrubs >0.5 m | 2 | <0.1 | 21 | 1.4 |
| *Rosa canina* | Trees and shrubs >0.5 m | 3 | 0.1 | 20 | 0.9 |
| *Rubus caesius* | Trees and shrubs >0.5 m | 1 | 0.3 | 1 | 0.2 |
| *Rubus plicatus* | Trees and shrubs >0.5 m | 2 | 0.1 | 1 | 0.2 |
| *Salix aurita* | Trees and shrubs >0.5 m | 1 | 0.6 | 1 | 0.2 |
| *Salix cinerea* | Trees and shrubs >0.5 m | 5 | 0.2 | 11 | 1.1 |
| *Sorbus aucuparia* | Trees and shrubs >0.5 m | 0 | 0 | 1 | <0.1 |
| *Solidago gigantea* | Invasive | 48 | 3.3 | 63 | 14.2 |
